# Outbreak of scrub typhus in odisha - an emerging threat

**DOI:** 10.1186/2197-425X-3-S1-A355

**Published:** 2015-10-01

**Authors:** SK Pattnaik, B Ray, S Sinha, A Mohanty, S Sahu

**Affiliations:** Apollo Hospitals, Bhubaneswar, India

## Introduction

Scrub typhus a zoonotic disease, caused by the intracellular parasite Orienta tsutsugamushi, transmitted by the bite of trombiculid mite. It is under reported in Odisha, may be due to lack of suspicion and / lack of specific diagnostic test.

## Objectives

To describe the diverse clinical and laboratory manifestations of scrub typhus patients admitted into the adult tertiary critical care unit at Apollo Hospitals, Bhubaneswar.

## Methods

All cases of febrile illness diagnosed as scrub typhus over a period of 9months from June 2014-Feb 2015 were analyzed. Diagnosis was based on the presence of the eschar and /or positive by IgM enzyme-linked immunosorbent assay (Sensitivity 93% and specificity 98%).

## Results

During the study period 40 cases of scrub typhus were detected and most of them presented with fever (100%), headache (80%), cough and dyspnea (65%). Eschar was detected in 12% cases. Mechanical ventilation (Invasive-18.5%, Non-invasive-81.5%) applied in 65% (n = 26) cases. All patients were managed with either doxycycline (n = 13; 32.5%) or doxycycline with azithromycin (n = 27; 67.5%). Among the patients 92.5% (n = 21) survived and 7.5% (n = 3) died.

## Conclusions

Scrub typhus has emerged as an important cause of febrile illness in Odisha. Empirical treatment with doxycycline is justified in endemic areas.

